# Prion protein insertional mutations increase aggregation propensity but not fiber stability

**DOI:** 10.1186/1471-2091-9-7

**Published:** 2008-03-17

**Authors:** Tejas Kalastavadi, Heather L True

**Affiliations:** 1 Department of Cell Biology and Physiology, Washington University School of Medicine, Box 8228, 660 South Euclid Avenue, Saint Louis, MO 63110, USA

## Abstract

**Background:**

Mutations in the *PRNP *gene account for ~15% of all prion disease cases. Little is understood about the mechanism of how some of these mutations in *PRNP *cause the protein to aggregate into amyloid fibers or cause disease. We have taken advantage of a chimeric protein system to study the oligopeptide repeat domain (ORD) expansions of the prion protein, PrP, and their effect on protein aggregation and amyloid fiber formation. We replaced the ORD of the yeast prion protein Sup35p with that from wild type and expanded ORDs of PrP and compared their biochemical properties *in vitro*. We previously determined that these chimeric proteins maintain the [PSI^+^] yeast prion phenotype *in vivo*. Interestingly, we noted that the repeat expanded chimeric prions seemed to be able to maintain a stronger strain of [*PSI*^+^] and convert from [*psi*^-^] to [*PSI*^+^] with a much higher frequency. In this study we have attempted to understand the biochemical properties of these chimeric proteins and to establish a system to study the properties of the ORD of PrP both *in vivo *and *in vitro*.

**Results:**

Investigation of the chimeric proteins *in vitro *reveals that repeat-expansions increase aggregation propensity and that the kinetics of fiber formation depends on the number of repeats. The fiber formation reactions are promiscuous in that the chimeric protein containing 14 repeats can readily cross-seed fiber formation of proteins that have the wild type number of repeats. Morphologically, the amyloid fibers formed by repeat-expanded proteins associate with each other to form large clumps that were not as prevalent in fibers formed by proteins containing the wild type number of repeats. Despite the increased aggregation propensity and lateral association of the repeat expanded proteins, there was no corresponding increase in the stability of the fibers formed. Therefore, we predict that the differences in fibers formed with different repeat lengths may not be due to gross changes in the amyloid core.

**Conclusion:**

The biochemical observations presented here explain the properties of these chimeric proteins previously observed in yeast. More importantly, they suggest a mechanism for the observed correlation between age of onset and disease severity with respect to the length of the ORD in humans.

## Background

The aggregation of various proteins is implicated in many diseases, including neurodegenerative disorders [1]. Many of the aggregating proteins form amyloid fibers that are the hallmark of diseases such as Alzheimer's, Parkinson's and Huntington's disease [2]. Amyloid fibers are also associated with prion diseases. The prion protein (PrP) can misfold and aggregate to form amyloid fibers, leading to neurodegenerative prion diseases [3,4]. The three primary means of acquiring prion diseases are as follows: 1) infection – can be transmitted by the ingestion of meat obtained from diseased animals, 2) spontaneous – occurs sporadically via unknown mechanism(s) and 3) inherited – mutations in the *PRNP *gene encoding PrP [4,5]. About 15% of prion disease cases are associated with mutations in the *PRNP *gene and ~85% of the cases are classified as sporadic [5]. A unique characteristic of prion diseases is that an aggregated form of the PrP protein can be infectious [6], however infectious forms of prion disease are rare in comparison to inherited and sporadic cases. Although the mechanism of pathogenesis of prion diseases is not well understood, this process does not necessarily result from a genetic mutation [3,7,8]. It is hypothesized that the aggregated form of PrP is infectious and acts as a template to change the native conformation of PrP and cause it to also aggregate [8]. Familial forms of prion diseases include Creutzfeldt-Jakob disease (CJD), Gerstmann-Sträussler-Scheinker syndrome (GSS), and Fatal Familial Insomnia (FFI). Several different mutations in *PRNP *are associated with prion diseases, but the mechanisms by which the various mutations cause disease are not always clearly understood [5]. In this study, we use a model system created to investigate some aspects of an inherited form of prion disease that results from insertional mutations in *PRNP *to cause diseases such as GSS and CJD [9].

PrP is a 23 kD protein that can be divided into two major domains. The C terminus is implicated in the formation of amyloid fibers in the infectious form of the disease [10,11]. The N-terminal domain is largely unstructured; however, some mutations in this domain are linked to disease and influence PrP fibrillization and amyloid propagation *in vitro *[12]. The N-terminal region contains an oligopeptide repeat domain (ORD) that consists of five repeats of an eight amino acid peptide with the consensus sequence PHGGGWGQ [13]. Expansion of the ORD by the addition of two to nine extra repeats causes a dominantly inherited form of prion disease [5,14-18]. The number of repeats present is inversely correlated, albeit weakly, with the age of onset of disease [19]. In addition, recombinant PrP peptides with expansions of the ORD display increased aggregation *in vitro *[20]. A transgenic mouse model of one such ORD expansion, Tg(PG14), in which the number of repeats in the ORD is increased to 14, develops a fatal neurodegenerative disorder [21,22]. The disease manifests spontaneously and the Tg(PG14) mice develop neurological illness that features ataxia, neuropathological abnormalities and the accumulation of PrP in the brain [21,23]. Furthermore, the ORD has been shown to bind many divalent cations such as copper, manganese and zinc [24,25]. It has been proposed that binding of these metals to the ORD may affect the structural properties of PrP and thereby affect disease progression [24]. Thus, many lines of evidence from human, mouse and biochemical studies illustrate a vital role for the ORD in some prion diseases.

Prion proteins also exist in yeast [26]. However, unlike the mammalian prion protein that causes disease when aggregated, yeast prions function as epigenetic factors of phenotypic inheritance [27-29]. One well-studied prion in yeast is the [*PSI*^+^] prion, whose protein determinant is Sup35p [30-32]. Sup35p is the *Saccharomyces cerevisiae *eRF3 (eukaryotic release factor 3) involved in modulating translation termination at stop codons in messenger RNAs. When Sup35p is in the prion conformation ([*PSI*^+^]), the aggregated protein is presumably no longer functional in translation termination, resulting in nonsense suppression [32,33].

Sup35p is a 76.5 kD protein that can be divided into three domains. The C-terminal domain is necessary and sufficient for the function of the protein in translation termination [30,34,35]. The amino-terminal region of Sup35p is considered the prion-forming domain (PFD) [30,35]. The minimum region required for maintenance of the [*PSI*^+^] prion is amino acids 1–93 [36,37], but this region is not sufficient to maintain different prion strains [38]. The N-terminal domain (N) is rich in glutamine and asparagine (Q/N) residues. The middle domain (M) is rich in charged amino acids [35]. Interestingly, the N-terminal domain of Sup35p contains an ORD that consists of five and a half repeats with the consensus sequence PQGGYQQYN [39] that is similar to the ORD in PrP. Deletion of even one repeat from the wild type Sup35 protein prevents efficient formation of the [*PSI*^+^] prion [36]. Expansion of the Sup35p repeats increases the frequency of conversion from the [*psi*^-^] to the [*PSI*^+^] state [39]. Previous studies have shown that the [*PSI*^+^] prion phenotype can be maintained *in vivo *when the Sup35p repeat region is replaced by the PrP repeat sequence[36,40]. The Sup35p repeats were replaced with five, eight, 11 or 14 PrP repeats to generate chimeras referred to as SP5 (Sup35 with 5 PrP repeats), SP8, SP11, and SP14, respectively [40]. All chimeras were capable of propagating a prion and maintained translation termination function in the non-prion state. Interestingly, the SP14 chimeric protein spontaneously converted cells from the non-prion [*sp14*^-^] state to the [*SP14*^+^] prion state at a frequency that is approximately four orders of magnitude higher than that of the spontaneous conversion of wild type Sup35p from the [*psi*^-^] to the [*PSI*^+^] state. This increase in the spontaneous conversion of the repeat-expanded chimera to the prion state may be due to an increase in the inherent propensity of the repeat-expanded proteins to aggregate and form amyloid fibers. However, the interpretation of the data is complicated by the requirement for the [*RNQ*^+^] prion for the enhanced induction *in vivo*.

Here, we take advantage of the chimeric Sup35-PrP prion protein system to study the effect of the PrP repeat expansions on the *in vitro *aggregation of the prion protein. The advantage of using the Sup35-PrP chimera lies in the ability to readily study properties of protein aggregation both *in vitro *and *in vivo*. By using the chimeric Sup35-PrP protein system, the effects of mutations associated with prion disease can be assessed by fiber formation *in vitro *and compared to their *in vivo *behavior in yeast, and vice versa.

In this study, we investigate the properties of the PrP ORD and disease-associated repeat expansion on amyloid fiber formation in an effort to distinguish between the properties of an ORD with non-pathological versus pathological repeat lengths. To this end, we have purified recombinant protein that encompasses the NM region of wild type Sup35p (Sup35NM) and that of chimeras with five (SP5NM), eight (SP8NM), 11 (SP11NM) or 14 (SP14NM) repeats of PrP precisely substituted for the Sup35p ORD and characterized their kinetic, morphological and biochemical properties.

## Results

### The expanded ORD of PrP decreases the lag phase of fiber formation

**Figure 1:**
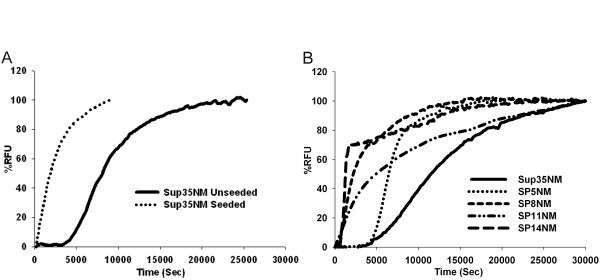
**Fiber formation of ORE chimeras is faster than that with the wild type ORD of PrP.** Recombinant protein was diluted from denaturant (120 ×) to 2.5 μM and fiber formation was followed by Th-T fluorescence. Graphs are plotted as %RFU (Relative fluorescence units) versus time. (A) Sup35NM unseeded (solid line) and Sup35NM seeded with sonicated preformed fibers (dotted line). Reaction was seeded with 2.5% (w/w) of preformed sonicated fibers. (B) Unseeded reactions Sup35NM (solid line), SP5NM (dotted line), SP8NM (short dashed line), SP11NM (dotted and dashed line) and SP14NM (long dashed line).

We purified the NM region of wild type Sup35p (Sup35NM) and chimeras with five (SP5NM), eight (SP8NM), 11 (SP11NM), and 14 (SP14NM) repeats. The purified recombinant proteins were used to assess the influence of ORD repeat expansions on amyloid fiber formation by monitoring the change in Thioflavin-T (Th-T) fluorescence over time. Wild type Sup35NM had a lag phase of ~5,000 seconds followed by a logarithmic growth phase (Fig. 1A). However, in the presence of seeds (sonicated preformed fibers 2.5% w/w), the lag phase was completely eliminated and fiber formation occurred rapidly (Fig. 1A) as reported previously [41]. We then assayed fiber formation of the chimeric proteins SP5NM, SP8NM, SP11NM and SP14NM. The formation of chimeric SP5NM fibers in an unseeded reaction showed a lag phase (~5,000 seconds; Fig. 1B) similar to that observed with wild type Sup35NM (Fig. 1A). The repeat-expanded chimeras SP8NM, SP11NM, and SP14NM had a negligible lag phase in unseeded reactions (Fig. 1B). Strikingly, the kinetics of fiber formation of the expanded ORD chimeric proteins in the unseeded reactions was similar to the kinetics of fiber formation of Sup35NM in the presence of pre-formed fiber seeds. In an effort to determine if the lack of lag phase associated with SP14NM fiber formation was simply due to seeds forming in the presence 6M GdHCl, we resuspended SP14NM protein in 8 M urea instead. When protein was diluted out from 8 M urea, we observed the same rate of fiber formation and no lag phase (data not shown).

**Figure 2:**
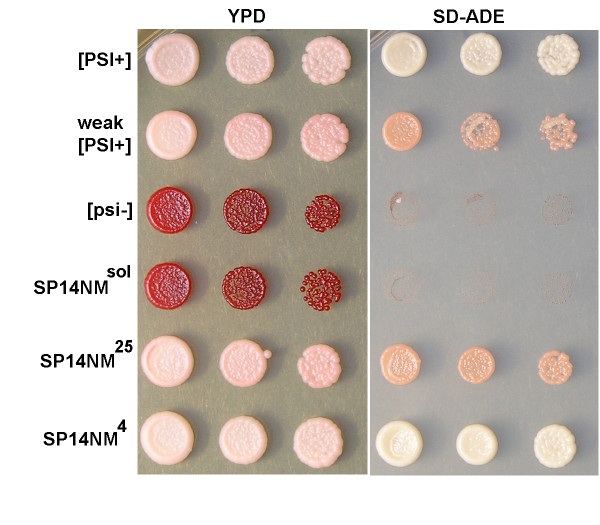
**Transformation of SP14NM protein into yeast induces [*PSI*^+^] only after fiber formation.** The [*PSI*^+^] phenotype was assayed by color on YPD, growth on SD-ADE and curability (not shown). Control protein transformations show that protein harvested from either strong or weak [*PSI*^+^] cells transformed in to [*psi*^-^] cells induces the corresponding prion phenotype while protein harvested from [*psi*^-^] cells does not induce the prion state. Soluble SP14NM protein (SP14NM^sol^) does not induce the [*PSI*^+^] prion phenotype prior to fiber formation. SP14NM protein was able to induce the [*PSI*^+^] prion phenotype after fiber formation either at 4°C (SP14NM^4^, strong strain) or 25°C (SP14NM^25^, weak strain).

Another widely used method that enables detection of infectious prion seeds is protein transformation. We tested for the presence of infectious seeds by transforming [*psi*^-^] yeast cells with purified SP14NM in 6 M GdHCl or SP14NM sonicated fibers. Cells transformed with SP14NM protein that was not allowed to form fibers did not acquire the [*PSI*^+^] phenotype (Fig. 2) in over 200 colonies tested, while 25%–30% of cells transformed with fibers acquired the [*PSI*^+^] phenotype (Fig. 2, data not shown). These data suggest that preformed seeds of SP14NM did not exist in the presence of 6 M GdHCl. However, formally the possibility exists that seeds are formed that are resistant to both denaturants and cause the reaction to appear as a seeded reaction. We observed that cells transformed with SP14NM fibers formed at 4°C generated mostly strong [*PSI*^+^] while 25°C yielded exclusively weak [*PSI*^+^] (Fig. 2). This illustrates the infectious nature of the *in vitro *formed SP14NM fibers. The appearance of strong and weak [*PSI*^+^] strains in cells that were transformed with fibers formed at different temperatures is in agreement with previously published results for Sup35NM [42]. The concept of yeast prion strain variants and their basis of structural variability has been studied extensively [42-45] and is presumably recapitulated with our repeat expansion chimeras.

**Figure 3:**
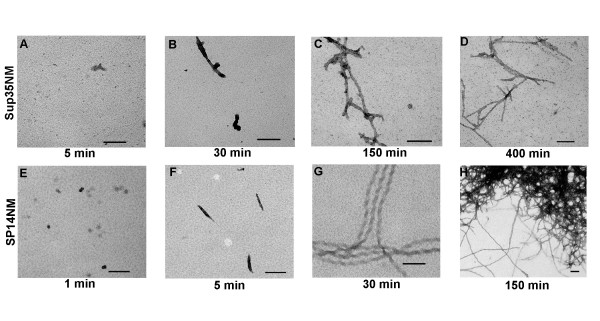
**Unseeded SP14NM fiber formation is faster than Sup35NM fiber formation as monitored by TEM.** The scale bar represents 100 nm. Sup35NM (5 μM) after (A) 5 min, (B) 30 min, (C) 150 min, (D) 400 min and SP14NM (5 μM) after (E) 1 min, (F) 5 min, (G) 30 min, (H) 150 min. Representative images are shown for each time point with the exception of (H) because the fibers were clumped, leaving the EM grid largely empty. See Figure 7A and 7E for comparisons to 24 hour time point.

**Figure 4:**
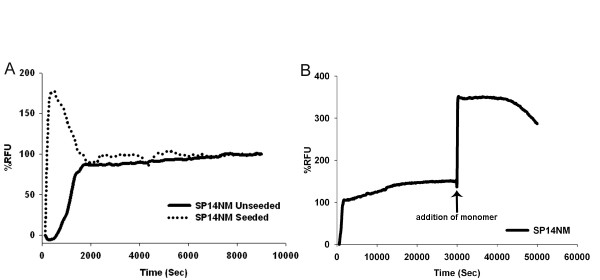
**The addition of preformed seeds causes a spike in SP14NM fiber formation.** Recombinant protein was diluted from denaturant (120 ×) to 2.5 μM and fiber formation was followed by Th-T fluorescence. Graphs are plotted as %RFU versus time and are representative of three experiments. (A) SP14NM unseeded (solid line) and SP14NM seeded (dotted line). (B) Another spike in Th-T fluorescence is observed after the addition of more monomer (2.5 μM) after the SP14NM fiber formation reached a plateau.

In order to verify that the repeat-expanded proteins form fibers faster than the wild type proteins, we visualized the formation of Sup35NM and SP14NM fibers over a time course using transmission electron microscopy (TEM). We were able to detect very few significant structures (above background) at five minutes with Sup35NM by TEM (Fig. 3A). Short fibers of Sup35NM became visible at 30 minutes and the formation of longer fibers was observed by 150 minutes (Fig. 3B, 3C). The TEM images obtained with Sup35NM at 400 minutes (Fig. 3D) were similar to images obtained after 24 hours of fiber formation, suggesting that the reaction had reached completion by 400 minutes. The TEM images supported the Th-T data which indicated that the reaction with Sup35NM plateaued by ~200 minutes (Fig. 1A). In contrast to wild type Sup35NM, we observed that the repeat-expanded SP14NM formed visible short fibers as early as five minutes and longer fibers by 30 minutes (Fig. 3E, 3F; compare to Fig. 4A unseeded reaction for expanded Th-T graph of early time points). TEM images of SP14NM suggest that fiber formation remains unchanged between 150 minutes (Fig. 3G) and 24 hours.

### Repeat-expanded prion protein cross-seeds the amyloid fiber formation of protein containing wild type repeat numbers efficiently

**Figure 5:**
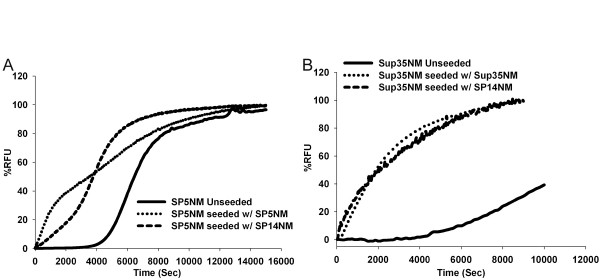
**Sup35NM and SP5NM are efficiently cross-seeded by SP14NM seeds.** Recombinant protein was diluted from denaturant (120 ×) to 2.5 μM and fiber formation was followed by Th-T fluorescence. Reactions were seeded with 2.5% (w/w) of preformed sonicated fibers as indicated. Graphs are plotted as %RFU versus time and are representative of three experiments. (A) Fiber formation of Sup35NM. (B) Fiber formation of SP5NM.

Prion diseases caused by oligopeptide repeat expansion (ORE) are inherited in an autosomal dominant manner. Patients who suffer from inherited prion disease typically express one copy of the repeat-expanded allele and one copy of the wild type allele [7,9]. Protein aggregates in these patients sometimes contain both the mutant and wild type protein [9]. One reason for the presence of both proteins in the aggregates could be that repeat-expanded PrP aggregates readily and cross-seeds the aggregation of the wild type protein. Therefore, we tested whether SP14NM seeds could enhance amyloid fiber formation of either Sup35NM or SP5NM monomers. We observed that SP14NM does cross-seed the aggregation of both Sup35NM (Fig. 5A) and SP5NM (Fig. 5B) efficiently. These data also support the previous suggestion that co-aggregation of wild type PrP and ORE PrP is a plausible factor in prion disease progression [9,20].

### Increasing repeat length in the ORD increases both incorporation of monomer and lateral association of amyloid fibers

Since the SP14NM amyloid fiber formation occurs almost instantaneously upon dilution from denaturant, we next asked whether there was any effect of adding seeds. We observed a sharp spike in fluorescence when SP14NM seeds were added to soluble SP14NM monomers (Fig. 4A), suggesting that fiber formation did occur by templating off of the pre-formed fiber seeds added. However, this spike was followed by a drop in fluorescence to a steady state level that was similar to that observed in the unseeded reaction. This was a very intriguing observation as no such drop in fluorescence was observed in the seeded Sup35NM fiber formation reaction (Fig. 1A). One possible reason for the tendency of the reaction to rapidly reach a steady state would be the inability of the fibers to incorporate additional soluble protein. In order to determine if the fiber ends were still competent for addition of soluble protein, we added fresh SP14NM monomer to a fiber formation reaction that had already reached the steady state level of Th-T fluorescence. The addition of SP14NM monomer at the reaction plateau caused another spike in fluorescence (Fig. 4B), suggesting that the fibers are still capable of adding soluble protein. Therefore, the plateau in fluorescence observed in seeded SP14NM fiber formation reactions was not likely due to an inability to incorporate additional monomer.

**Figure 6:**
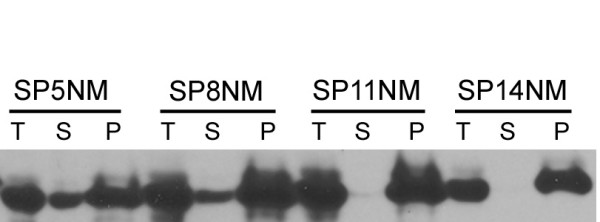
**Soluble protein is incorporated into fibers more efficiently in chimeras with increased number of repeats in the ORD.** Fiber formation reactions (5 μM) were separated into supernatant (S) and pellet (P) fractions by high speed centrifugation and analyzed by SDS-PAGE and western blot using α-Sup35 antibodies. Soluble protein remains in the supernatant fraction and the fibers are found in the pellet fraction. (T) is the total reaction prior to centrifugation.

Alternatively, the initial spike observed in the seeded SP14NM reaction could arise due to fibers forming at the instant the seed is added, while the ensuing drop in fluorescence could result from dissociation of protein from the fibers. The plateau would then be a result of the reaction reaching an equilibrium state. To address this hypothesis, we assessed the amount of soluble protein at the end-point of fiber formation using a centrifugation assay. First, all chimeras were allowed to form fibers overnight. Then we separated the amyloid fibers from the soluble protein by centrifugation and examined the protein in the fractions by SDS-PAGE and western blot. We observed that the amount of remaining soluble protein decreased as the number of repeats increased (Fig. 6). The amyloid fiber formation reaction of either SP5NM or SP8NM showed a considerable amount of soluble protein after an overnight incubation, as seen by the protein in the supernatant fraction. However, there was little and no detectable protein in the supernatant from either SP11NM or SP14NM fiber formation reactions, respectively. The lack of soluble protein remaining in the SP14NM fiber formation reaction negated the hypothesis that the drop in fluorescence in seeded SP14NM reactions occurred as a result of dissociation of protein from the amyloid fibers over time.

**Figure 7:**
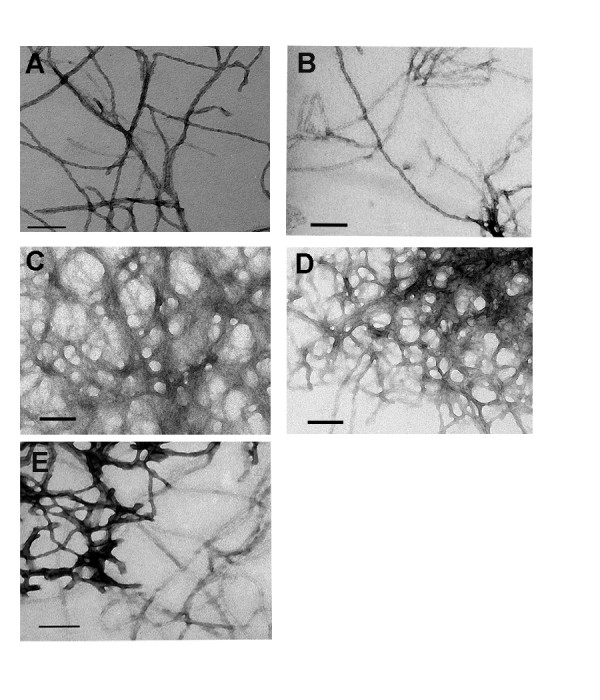
**The morphology of amyloid fibers formed from protein containing pathological versus non-pathological repeat lengths is distinct.** Unseeded fiber formation reactions (5 μM) were imaged by TEM after 16 hours. The length of scale bar represents 100 nm. (A) Sup35NM and (B) SP5NM have uniform dispersed fibers. (C) SP8NM, (D) SP11NM and (E) SP14NM have clumped fibers.

This led us to hypothesize that the spike and drop pattern of Th-T fluorescence seen in seeded SP14NM fiber formation reactions might be due to exclusion of Th-T binding over time. The increase in fluorescence occurs as Th-T initially binds the rapidly forming fibers and the drop may result from Th-T being occluded from the fibers as the reaction progresses. In order to address this hypothesis, we returned to TEM in order to examine the morphology of the fibers of wild type Sup35NM and the chimeric proteins. Interestingly, TEM revealed a morphological difference between the Sup35NM and SP14NM fibers. We investigated the morphology of fibers after 24 hours of fiber formation of all the chimeric proteins. We observed that fibers formed from proteins with the wild type number of repeats, Sup35NM and SP5NM did not have a clumped morphology (Fig. 7A, 7B). However, fibers formed from SP8NM, SP11NM, and SP14NM proteins all exhibited a highly clumped morphology by TEM (Fig. 7C, 7D, 7E). The repeat-expanded ORD chimeric proteins showed an increased lateral association of fully formed fibers as compared to the fibers formed with either Sup35NM or SP5NM. Furthermore, when fibers formed overnight by SP14NM were sonicated, there was an increase in Th-T fluorescence (data not shown). This suggests that sonication disrupted the clumps and once again increased the surface area for Th-T to bind the fibers. Such an increase in fluorescence was not observed when the same experiment was done with fibers formed from either Sup35NM or SP5NM (data not shown). These data support the hypothesis that the difference in Th-T fluorescence is due to the morphological differences between the fibers. As the fibers laterally associate and form large clumps, sites that were previously available for Th-T binding may be unable to bind to Th-T as they interact with other fibers. Such Th-T occlusion would cause a drop in fluorescence.

### Prion protein repeat expansion allows fiber formation in the presence of denaturant

**Figure 8:**
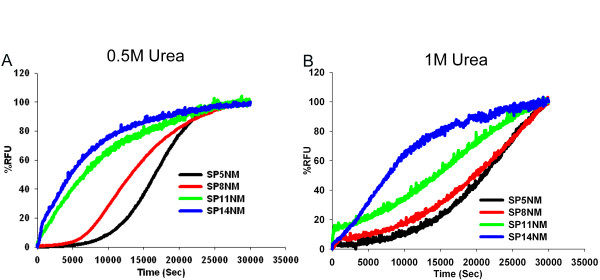
**Expansion of the ORD enhances fiber formation in the presence of the denaturant urea.** Recombinant protein was diluted from denaturant (120 ×) to 2.5 μM and fiber formation was followed by Th-T fluorescence. Fiber formation of chimeric proteins SP5NM, SP8NM, SP11NM and SP14NM in (A) 0.5 M Urea FFB (B) 1 M Urea FFB is shown. Data in the graphs are representative of two experiments.

In the absence of denaturant, fiber formation of Sup35NM and SP5NM exhibits a lag phase of ~5,000 seconds (Fig. 1B). However, SP8NM, SP11NM, and SP14NM form fibers instantaneously without an appreciable lag phase (Fig. 1B). This suggests that expansion of the repeat region increases the propensity of the proteins to aggregate. However, with these data we were unable to discern how the propensity to aggregate increases with the change in repeat length. There may be a gradual increase in aggregation propensity with an increase in repeat number or there may be a critical number of repeats after which the protein forms fibers without a lag phase. To further characterize the effect that increasing repeat number has on protein aggregation, we assembled fibers in the presence of increasing concentrations of urea. In the presence of 0.5 M urea, fiber formation of unseeded SP5NM had an extended lag phase of approximately 10,000 seconds (compared to 5,000 seconds in the absence of urea). Unseeded SP8NM had a lag phase of approximately 5,000 seconds (compared to no lag phase without urea) while the lag phases of fiber formation of either SP11NM or SP14NM were not affected by 0.5 M urea (Fig. 8A). When the urea concentration was increased to 1 M in fiber formation reactions of SP5NM and SP8NM, the Th-T fluorescence did not reach a plateau within 30,000 seconds (Fig. 8B), suggesting that fiber formation did not reach completion within the same time frame as in the absence of denaturant (Fig. 1B). SP11NM also formed fibers at a much slower rate in the presence of 1 M urea (Fig. 8B). However, the lag phase of SP14NM fiber formation was not affected by 1 M urea. In the presence of 2 M urea, we did not detect fiber formation with any of the proteins by Th-T fluorescence (data not shown). These results suggest that the propensity of the chimeric prion proteins to aggregate is a function of the number of repeats in the protein; as the number of repeats increase, the propensity of the proteins to aggregate gradually increases.

### Prion protein repeat expansion does not enhance stability of amyloid fibers

**Figure 9:**
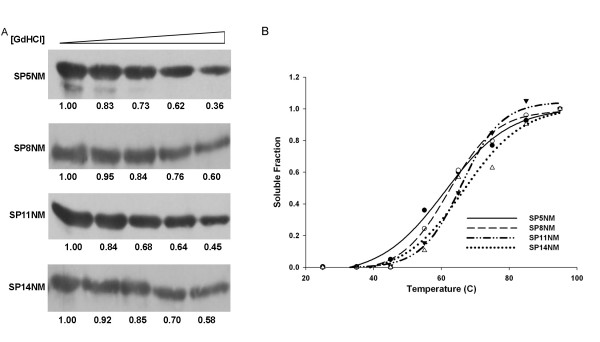
**The stability of the prion protein fibers is not enhanced by expansion of the repeat domain.** (A) Fibers were formed overnight, treated with GdHCl (0 M, 0.5 M, 1.0 M, 1.5 M and 2 M) denaturant and then separated into soluble and pellet fractions by centrifugation (16,000 × g). Samples were analyzed by SDS-PAGE and western blotted using α-Sup35 antibodies. Bands were quantified using ImageJ software. (B) Fibers of SP5NM (●), SP8NM (○), SP11NM (▼) and SP14NM (△) were formed overnight, treated across a temperature gradient in the presence of 2% SDS. Following SDS-PAGE and western blot, gel bands were quantified using ImageJ software and and best fit lines were graphed using SigmaPlot software.

ORD-expanded proteins aggregate to form amyloid fibers much faster than the wild type protein. An interesting question that arises from this is: Do the amyloid fibers formed by ORD-expanded proteins differ in stability? That is, does the expansion of the ORD enhance fiber stability? It is possible to imagine that mutations that cause proteins to have a greater on-rate for fiber formation may have a slower off-rate and thereby make the amyloid fiber more stable. One way of measuring the stability of amyloid fibers *in vitro *is to examine their denaturation profiles. Amyloid denaturation profiles can be evaluated by treating the fibers with increasing concentrations of denaturant and determining the concentration at which half of the protein is resolubilized. We measured the denaturation profiles of the fibers formed from all of the chimeras by determining the amount of protein remaining aggregated after treatment with increasing concentrations of GdHCl. Using this method, we were able to determine the concentration of GdHCl at which the protein present in the pellet was depleted by approximately 50%. Interestingly, approximately 50% of the pelletable protein was depleted for all the chimeras at GdHCl concentrations between 1.5 – 2 M (Fig. 9A), suggesting that the ORD expansion does not enhance the stability of fibers. We also measured stability of the chimeras by monitoring Th-T fluorescence after treatment with increasing concentrations of GdHCl and observed that the loss of Th-T fluorescence was similar for all chimeric proteins and mirrored the centrifugation results (data not shown). Next, we examined fiber stability in a temperature solublization assay. We treated fibers across a temperature gradient from 25°C to 95°C at 10°C intervals in the presence of 2% SDS and visualized soluble protein by western blot. We observed that 50% of the protein becomes soluble between 55°C – 65°C for all the chimera fibers (Fig. 9B). Taken together, these data suggest that the stability of the amyloid fibers formed by the chimeric proteins is the same, irrespective of the ORD repeat length.

## Discussion

In this study, we characterized the biochemical properties of a set of chimeric prion proteins wherein the ORD of Sup35p was replaced with that of PrP. The chimeric prion proteins were created by substituting the endogenous Sup35p ORD with the repeat domain of PrP containing five, eight, 11 and 14 oligopeptide repeats [40]. The repeat-expanded proteins show a remarkable set of properties that highlight their enhanced ability to aggregate and form amyloid fibers *in vitro*. These data agree with work done by others in which recombinant PrP (rPrP) with ORD expansions exhibit an enhanced ability to form amyloid fibers with increasing number of repeats [20,46]. Our data also support previous work done with transgenic mice (Tg(PG14)) that express PrP harboring nine additional octapeptide repeats. These mice manifest a spontaneous form of prion disease [21]. Although the spontaneous form of the disease in the Tg(PG14) mice is not infectious, the protein aggregates and the animals display many of the histopathological hallmarks that are characteristic of prion diseases in mammals [23].

In humans, there is a high degree of heterogeneity with respect to the age of onset and disease progression in people that have mutations in the *PRNP *gene [19,47,48]. There is a weak inverse correlation between the number of repeats and the age of onset of disease [19]. Further, repeat-expanded proteins have also been proposed to interact with the wild type PrP protein. Examination of amyloid plaques from patients with one copy of wild type PrP and one copy of mutant PrP demonstrated the presence of both the wild type and the mutant forms of the protein in the same plaques [9]. It has also been shown that mixing repeat-expanded rPrP with wild type rPrP enhances fiber formation of wild type rPrP [20]. These data suggested that there may be co-aggregation or cross-seeding between the mutant and wild type proteins. Our data also supports the cross-seeding model of disease progression since SP14NM shows efficient cross-seeding of both Sup35NM and SP5NM.

We observed that an increase in the number of repeats enhanced the ability of the protein to form fibers to the extent that the lag phase observed during most amyloid fiber formation reactions was lost. In addition, the proteins harboring repeat expansions had an enhanced ability to form fibers in the presence of denaturant. Interestingly, comparing our data to a recent study [25] suggests that the presence of heterogeneous repeats, that is, a combination of one Sup35p repeat with eight repeats from the PrP protein, forms amyloid fibers slower than having eight repeats from PrP only (Fig. 1B). This suggests that the enhanced amyloid formation with repeat expansion is affected by repeat homogeneity. Strikingly, our data suggests seeding of fibers with proteins having different (Sup35p or PrP) repeats appears to be as efficient as seeding with proteins that harbor same repeats (Fig. 5B).

Previous work by Serio *et al. *suggested a nucleated conformational conversion model of amyloid fiber formation of Sup35NM [49]. An important aspect of this model is that the rate limiting step for fiber formation, manifested as the lag phase, is the conformational conversion of the nucleus to establish a competent seed. One implication of this model is that rate of fiber formation is not linearly dependent on initial concentration of protein. Serio *et al. *showed that the lag phase of Sup35NM fiber formation was not affected significantly by the initial protein concentration. We observed that the lag phase of SP14NM fiber formation, albeit an extremely short lag phase, is also not affected by initial protein concentration. In fact, even when the concentration of SP14NM protein was only 0.25 μM in unseeded fiber formation reactions, the lag phase of fiber formation was identical to that of 5 μM protein (data not shown). Our kinetic data suggest that the SP14NM nucleus has an enhanced ability to undergo conformational conversion to the extent that the lag phase of fiber formation is virtually eliminated. As previously suggested [43], one mechanism that could explain the decrease in lag phase for fiber formation is that the expanded ORDs have transient β-sheet structures that are formed due to intramolecular contacts, which may reduce the number of intermolecular contacts required to obtain a stabilized structure to nucleate fiber formation. This would suggest that if a monomer can undergo the appropriate conformational changes and maintain that conformation through intramolecular contacts, then the protein may not need to form oligomers in order to form fibers. Currently, it is unclear if the growth of amyloid fibers in general occurs through linear addition of monomers or intermediate fibers [50,51]. Different amyloidogenic proteins may have distinct mechanisms for the growth of amyloid fibers [52]. Work done by others suggests that fibers formed by Sup35NM occurs by monomer addition [51], however, in the case of the A-beta peptide, an oligomeric intermediate may be necessary for fiber growth [53,54]. One current model for the growth of poly-Q peptides suggests that a single molecule, after undergoing conformational conversion, may act as a nucleus capable of templating the addition of monomers, thereby resulting in the growth of amyloid fibers [50]. Therefore, it is plausible that for PrP molecules with expanded ORDs, the mechanism of amyloid fiber assembly and growth changes such that the formation of an oligomer for initiation or growth of fibers is no longer required.

In addition to the ability of repeat-expanded proteins to form a competent seed more readily, a difference in the pathological and non-pathological repeat expansions may be in the efficiency with which monomer is added to the fibers. Our data from the centrifugation assay suggests that the ability of the conformationally-converted nucleus to incorporate soluble protein increases as the number of repeats increase. Currently, a debatable idea in the amyloid field is the existence of a critical concentration in amyloid fiber formation reactions. It has been suggested that the addition of monomer to the fibers may be reversible [55]. Therefore, by assaying the amount of monomer remaining at the end point, we could potentially determine the relative critical concentrations [56]. As such, our data from the centrifugation assay may also suggest that as the number of repeats in the ORD increases, the critical concentration decreases. However, we do not know if these reactions are in fact reversible.

Since the repeat-expanded proteins form fibers faster and incorporate monomer more efficiently, we hypothesized that the fibers formed may also be more stable. However, the increased repeat length did not alter amyloid stability as assessed by denaturation in GdHCl or resolubilization by heat treatment. We observed no significant shift in the concentration of GdHCl or the range of temperature that resolublized ~50% of the fibers formed by the chimeras. This suggests that, irrespective of which mutant repeat expansion protein is found from patient to patient, the clearance of the aggregates might be equally challenging to the cellular machinery. One reason for the lack of difference in stability might be that the amyloid core of the fibers does not change significantly between fibers formed with the various repeat-expanded monomers. We crudely assessed changes in the amyloid core of the fibers by treating the fibers with various proteases and determining the pattern of protease resistant fragments. We observed similar patterns of protease resistant fragments for all of the ORD-expanded proteins (chymotrypsin, V8, proteinase K; data not shown). This suggests that the amyloid core of all the fibers, regardless of the monomer used to form the fibers, might be the same. In order to conclusively determine the amyloid core of all these proteins, however, more sensitive biophysical assays are required.

From our previous *in vivo *experiments [40], we noted that SP14NM can maintain the [*SP14*^+^] prion phenotype in weak and strong variants similar to the wild type [*PSI*^+^]. However, a very unique characteristic of the [*SP14*^+^] prion lies in its ability to interconvert between weak and strong variants at a high frequency. One explanation for the ability of [*SP14*^+^] to interconvert variants readily may be that the amyloid core does not change significantly between the two variants. Therefore, the protein would readily be able to adopt either conformation and thereby switch variants at a high frequency. This differs from wild type Sup35p, where weak and strong strains of the [*PSI*^+^] prion have different amyloid cores [43,45]. The recent study by Toyama *et al. *shows that the first 40 amino acids of Sup35p are part of the amyloid core for [*PSI*^+^] strains [45]. Interestingly, data published by our lab demonstrates that the repeat expansion can overcome the requirement of the first 40 amino acids in prion propagation [40]. Taken together, and discounting the obvious technical differences between all of these studies, the data suggest that there may be multiple ways to generate prions and structural variants [40,43,45]. The strain variants observed in yeast can be used as a model to study the phenotypic heterogeneity that is exhibited by strains of prion disease. The ability of [*SP14*^+^] prion to interconvert between variants rapidly may provide an avenue to investigate the mechanisms underlying the high degree of phenotypic heterogeneity observed with prion diseases that arise due to repeat expansions in *PRNP*.

## Conclusion

In summary, biochemical characterization of the repeat expanded proteins demonstrated that the proteins harboring repeat expansions of pathological length have a greater propensity to aggregate and have a considerably shorter lag phase in fiber formation. The morphology of the fibers is also different between the non-pathological and pathological repeat expansions. Amyloid fibers formed with repeat-expanded proteins clump into large aggregates, whereas the fibers formed by proteins that do not have repeat expansions do not laterally associate to the same extent. Chimeras with the disease-associated repeat expansions proved to be more efficient at converting soluble protein into the aggregated state than the proteins containing wild type repeat numbers. In addition, the fibers formed from all proteins showed similar denaturation and solubilization profiles when treated with guanidine hydrochloride (GdHCl) or heat. Together, our data suggest that the expansion of the ORD in PrP results in an increased propensity of the protein to convert from the native conformation to an aggregated conformation, but does not alter the stability of the amyloid fibers formed.

## Methods

### Protein expression and purification of recombinant proteins

Sup35NM was purified as reported previously [57]. SP5NM, SP8NM, SP11NM and SP14NM were subcloned (from [40]) into the vector pET22. Protein was expressed in BL21(DE3)pLysS *Escherichia coli *cells grown in CircleGrow^® ^medium containing chloramphenicol (34 μg/ml) and ampicillin (100 μg/ml). Protein expression was induced with 1 mM IPTG, at OD_600_~0.6 at 24°C for four and a half hours. The bacterial pellet was resuspended in buffer A (8 M urea, 10 mM Tris-HCl, pH 7.5) and gently agitated at room temperature for 20 minutes. The cell debris was removed by centrifugation for 30 minutes at 17,000 rpm in a Sorvall SS-34 rotor. The supernatant was loaded onto a Q-Sepharose (GE HealthCare) column and the protein was eluted with a linear gradient of sodium chloride (0 – 1 M NaCl). Fractions containing the protein were loaded onto a hydroxyapatite column (BIO RAD) equilibrated with buffer C (8 M Urea, 5 mM KPO_4_, pH 6.8). The protein was eluted with a linear gradient of potassium phosphate (5–500 mM). Fractions containing the protein were determined by SDS-PAGE and coomassie staining. The fractions containing the protein were pooled and dialyzed against Buffer A and stored in methanol at -80°C.

### Amyloid Fiber Formation Kinetics

Recombinant protein was methanol-precipitated and resuspended in 6 M GdHCl. Protein concentration was determined by measuring the OD at 280 nm. The protein was diluted 120-fold in FFB buffer (150 mM NaCl, 5 mM KPO_4_, pH 7.5) for fiber formation assays. Fiber formation was followed by monitoring Thioflavin-T binding (100-fold excess). Thioflavin-T fluorescence was continuously measured using PTI Quantamaster spectrofluorometer (Photon Technology International, Inc., Santa Clara, CA).

### Electron Microscopy

Samples of fibrillar Sup35NM, SP5NM, SP8NM, SP11NM and SP14NM were allowed to settle onto freshly glow-discharged 200 mesh carbon-formvar coated copper grids for 5 minutes. Grids were then rinsed twice with water and stained with 1% uranyl acetate (Ted Pella) for one minute. Samples were viewed on a JEOL 1200EX transmission electron microscope (JEOL USA).

### Centrifugation Assay

Amyloid fibers were formed by incubating recombinant protein diluted 120-fold in FFB overnight at room temperature while rotating end-over-end. The reaction was separated into pellet and supernatant fractions by centrifugation (16,000 × g, 20 minutes). The total, supernatant and pellet fractions were separated by SDS-PAGE and analyzed by western blot using α-Sup35 antibodies.

### GuanidineHydrochloride (GdHCl) denaturation profile

Recombinant protein was diluted 120-fold in FFB and incubated at room temperature on a rotator overnight. The fibers were treated with increasing concentrations (0–2 M) of GdHCl for 30 minutes. The treated fibers were then separated into supernatant and pellet fractions by centrifugation (16,000 × g, 20 minutes). The pellet fractions were separated by SDS-PAGE and analyzed by western blot using α-Sup35 antibodies. The band intensities were quantified using ImageJ software.

### Temperature resolubilization assay

Recombinant protein was diluted 120-fold in FFB and incubated at room temperature on a rotator overnight. These fibers were then incubated across a temperature gradient (25°C – 95°C, 10°C intervals) for 5 minutes in the presence of 2% SDS. After the heat treatment, the samples were analyzed by SDS-PAGE and western blot. The amount of protein that entered the gel was determined by quantifying the bands using ImageJ software.

### Protein transformation

Protein transformation into 74-D694 yeast strain was conducted as described in Ref. [58].

## Abbreviations

IPTG: isopropyl-beta-D-thiogalactopyranoside, ORD: Oligopeptide Repeat Domain, ORE: Oligopeptide Repeat Expansion, PFD: Prion Forming Domain, PrP: Prion Protein, SDS-PAGE: Sodium Dodecyl Sulphate-PolyAcrylamide Gel Electrophoresis, TEM: Transmission Electron Microscopy, Th-T: Thioflavin-T.

## Authors' contributions

TK and HLT conceived and designed the study. TK conducted the experiments and together with HLT analyzed the results. TK and HLT drafted the manuscript. TK and HLT have read and approved the final manuscript.
